# The design and initial patient evaluation of an integrated care pathway for faecal incontinence: a qualitative study

**DOI:** 10.1186/s12913-015-1108-5

**Published:** 2015-10-01

**Authors:** Craig John Rimmer, Kathryn Ann Gill, Sheila Greenfield, George Dowswell

**Affiliations:** Sandwell and West Birmingham Hospitals NHS Trust, Birmingham, UK; University of Birmingham, Birmingham, UK

**Keywords:** Faecal incontinence, Integrated care pathway, Patient evaluation, Qualitative study

## Abstract

**Background:**

Faecal incontinence is a common, distressing and debilitating condition which remains largely hidden, leading to social isolation and loss of confidence. Patients with faecal incontinence experience delays in accessing appropriate treatment services due to embarrassment and lack of enquiry from primary care health professionals. Despite the publication of three government documents related to continence services in the last decade, these services are still fragmented with asynchronous delivery and poor inter-professional integration.

The aim of the study was to describe a novel integrated care pathway for the management of faecal incontinence and examine the experiences of patients with faecal incontinence in relation to this pathway.

**Methods:**

A focus group (eight participants) and narrative, qualitative individual interviews (five participants) were used to explore the views of patients with faecal incontinence, relating to access and quality of incontinence services and the new integrated care pathway. Emerging themes were identified from the transcribed focus group and interviews via the thematic analysis method.

**Results:**

The concept of an integrated care pathway is attractive for increasing accessibility, streamlining of the patient pathway and providing a dedicated service for the management of faecal incontinence. Patients’ initial experiences of the pathway are positive.

**Discussion:**

A new ICP was developed and the initial patient evaluation of it was positive. Service users made various suggestions how the FI pathway could have been improved. The issues that patients were most concerned about were access to continence services, GP awareness of continence services and prompt, effective management of their condition. This service was set up within the pelvic floor dysfunction unit with BFNS and an integrated community continence team. The authors are aware that this is not a standard service setup across the country. The fact that it may be uncomfortable for patients to talk about their condition may have led to potential bias when discussing their beliefs or experiences. As with most qualitative studies, our aim was to identify a range of experiences rather than define our participant sample as being representative. Our participant sample was diverse in the key characteristics but a longitudinal study may reveal further important aspects of an ICP for FI.

**Conclusions:**

An integrated care pathway for faecal incontinence appears to have potential to address the long-standing service delivery issues that have blighted continence services historically.

**Funding: The authors declare no external funding was associated with this study Electronic supplementary material:**

The online version of this article (doi:10.1186/s12913-015-1108-5) contains supplementary material, which is available to authorized users.

## Background

Faecal incontinence (FI), the involuntary loss of liquid or solid stool that is a social or hygienic problem, is a common and socially isolating healthcare problem. The estimated prevalence of FI varies widely, from 1.5 to 50 % [1]. Reasons for the wide variation in prevalence estimates include the definition of incontinence used, the clinical setting (i.e. nursing home or community), age of patients and the influence of social stigma on the patient, which can lead to under reporting of the condition [2]. The cause of FI is often multifactorial with anal sphincter weakness, pudendal neuropathy, impaired anorectal sensation, impaired rectal accommodation and incomplete evacuation all potentially contributing to the pathogenesis of FI [3, 4].

Whilst the treatment of FI requires sophisticated and co-ordinated management across a number of service boundaries, in reality the care provided is often disjointed, with patients and their carers obliged to navigate complex, fragmented systems over extended periods, with poor access to the social, psychological and specialist support needed to address their specific needs [5, 6]. Individuals who have a negative experience at their first attempt at seeking help will often be discouraged from seeking help again [7]. For this reason it is important to ensure that patients are identified and treated with evidence based practice in an efficient, streamlined 'seamless' manner in order to achieve the best possible outcomes from conservative management and to ensure that appropriate specialist care is available for those who require it.

Several National Institute for Health and Clinical Excellence (NICE) and Department of Health documents advocate the use of an integrated continence service involving a multidisciplinary team of healthcare professionals possessing the relevant skills and expertise to manage patients with FI [8–10]. These documents have drawn attention to the fact that the majority of FI and continence services, in general, remain fragmented, with poor access and variable outcomes [8–10].

Service delivery specifically for sufferers of FI, has been beset by a number of problems that have prevented the implementation of the recommendations of various Government papers and NICE guidelines in this area. Amongst these have been:

- Poor acknowledgement by sufferers of the problem, and lack of awareness that help is available [11–13]
- Lack of recognition of the problem by clinicians and/or awareness of new, more effective techniques [14]
- Changes to working practices including increased workload for community health care professionals [15]
- Poorly developed services or lack of awareness of existing services amongst clinicians [16]

Thus, the successful management of patients with FI clearly requires well-organised, coordinated health care support. A potential solution for this could be provided by the introduction of an integrated care pathway.

Integrated care pathways (ICP) have gained increasing popularity within the United Kingdom as a tool for managing clinical processes and patient outcomes in the last 30 years [17]. ICPs are multidisciplinary plans that predict the course of events in the treatment of patients with similar problems. The aim of an ICP is to enhance the quality of care by improving patient outcomes, promoting patient safety, increasing patient satisfaction and optimising the use of resources [18].

A West Midlands Trust has recently implemented a novel ICP for the management of patients with FI. The Trust already had a pelvic floor dysfunction service set up by the colorectal team that managed patients with chronic constipation, rectal evacuatory disorders and FI. The multidisciplinary team agreed that patient access to the service, referrals into the service, the triage process and the management of people with FI could all be improved locally based on the publications by NICE [8] and the Department of Health [9]. Following further dialogue between the multidisciplinary team, management within the Trust and the local Primary Care Trust, it was felt that the improvements within the service could be achieved through the development of an integrated service across primary and secondary care. The combined team then developed a proposal for an integrated care pathway (ICP) for the management of FI that was intended to underpin such a service and the implementation of this new, integrated model for the management of FI was commenced at the latter end of 2012. We carried out a qualitative study to describe the experiences of patients suffering from FI with regards to the original services provided and access to those services. In this article we also give a description of the new ICP for FI and report initial patient experiences of the new ICP.

### History, context and the implementation of an ICP for FI

In 2008, a pelvic floor dysfunction service was developed at a West Midlands NHS Hospital Trust, following the appointment of a new Consultant Colorectal Surgeon with a specialist interest in pelvic floor disorders. Provision of this service had previously been very limited. A pelvic floor dysfunction service to identify, assess and manage patients suffering from chronic constipation and FI was established. By 2011, the new service provided patients with a complete service based solely within the Trust, where they could be assessed and managed without needing to travel to other hospital trusts for any diagnostic studies, which had not been the case previously. The clinical outcomes of patients and patient reported outcome measures (PROMS) were continuously measured throughout the development of the pelvic floor dysfunction service and large improvements in both types of outcomes were made, to a standard whereby outcome data was being presented to national and international scientific surgical and nursing meetings [19].

Following the publication of a number of government documents related to continence [8, 9] the lead Consultant and multidisciplinary team (MDT) decided to focus on improving the pelvic floor dysfunction service. The MDT (consisting of the secondary care and community healthcare professionals managing patients with FI, and GPs) agreed that patient access to the service, referrals into the service and the triage process could all be improved locally by modelling the service on the publications and guidance issued by NICE [8] and the Department of Health [9]. Following further dialogue between the MDT and the local Primary Care Trust, it was felt that the improvements within the service could be achieved through the development of an integrated service across primary and secondary care. This approach was taken to attempt to rectify the long-standing issues highlighted by the Good practice for continence services report [9], and the National Audit of Continence Care [20]. Alongside these publications, the All Party Parliamentary Group for Continence Care Report on Cost effective commissioning for continence care [21] was used to help with the process of service redesign, especially for the service leads in primary and secondary care. A proposal was developed for a new ICP for the management of patients with FI that was subsequently implemented in October 2012.

There were three aims of the new ICP: improving accessibility and awareness of patients with FI, aiming to get patients seen by the appropriate healthcare professional at the appropriate time and ensuring these patients are seen in an appropriate location. To achieve these aims the ICP needed to have certain elements within it, including GP and patient education to improve awareness, a clearly defined referral pathway for GPs, in conjunction with a clinical pathway to ensure triage, assessment and management of patients was consistent. All of this was to be delivered using an electronic pathway document for healthcare professionals, with patients having a handheld paper document. With regard to GP education, this was delivered by the lead Consultant and bowel function nurse specialist (BFNS), to individual GP practices with all of the GPs within the practice being present during the session. This session was typically arranged during either a practice meeting or GP education session and involved a short presentation on the background of the pelvic floor dysfunction service, the ICP itself, the ICP document (handheld and electronic) and the referral process.

**Fig. 1:**
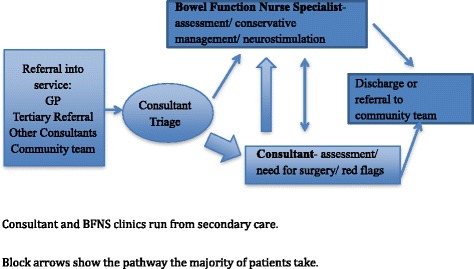
Traditional pathway

**Fig. 2:**
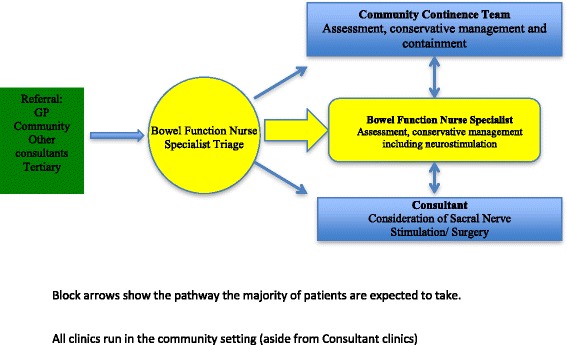
Integrated care pathway for faecal incontinence

When comparing the ‘traditional’ pathway (Fig. 1) to the new ICP pathway (Fig. 2) there are three key differences. Firstly, was the location of care. All clinic visits (aside from consultant clinics) took place in the community setting, run either by the BFNS or the community continence team (CCT). There were two large GP practices that the clinics were run from within the local geographical area. The second difference was the change in triage process from consultant triage to BFNS triage.

The third change was the inclusion of the CCT within the central pathway. Previously, the CCT had been very much a last resort following either completion of or maximal treatment in the secondary care setting. With the inclusion of the CCT, the ICP ensured further integration of working. The change in triage process aimed to change the patient flow. Patient flow changed from all patients having to be seen initially by the consultant to being reviewed initially by the BFNS/CCT or consultant following BFNS triage. Following review by these two members of the team, if a consultant review was required then patients were referred or discussed with the consultants via the MDT meeting within the pelvic floor dysfunction service.

**Fig. 3:**
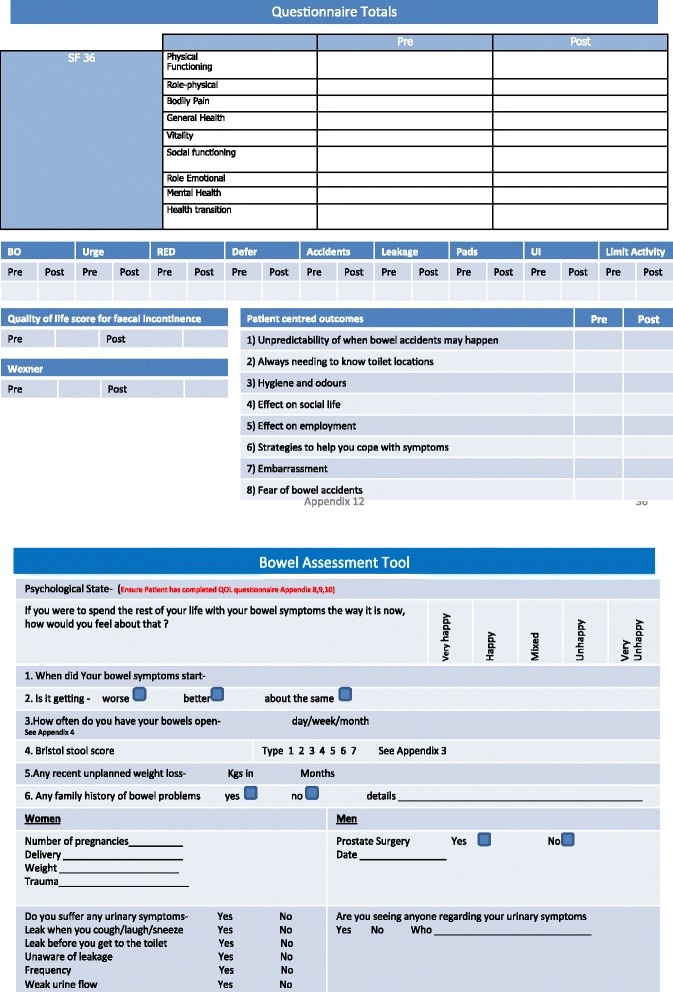
Examples of integrated care pathway document

Elements were also added to continence care. The introduction of a patient handheld document alongside an electronic version, which was linked with primary and secondary care, helped to give patients and healthcare professionals a permanent record of their care. This document possessed all of the assessment tools used by the service as well as patient instructions on how to perform some of the conservative management techniques (see Fig. 3). There have been no changes to treatment modalities for patients on the ICP when compared to the ‘traditional’ pathway. As the service was already evidence based and clinical audits had shown good patient outcomes, the treatment modalities were not expected to change.

The aim of this study was to identify the perspectives of patients with FI in relation to a new ICP in comparison to their previous experience with continence services.

## Methods

The study was performed in the West Midlands region, using a focus group of local community continence user group patients and interviews with individual patients who attended the new ICP clinic. University of Birmingham ethical approval was obtained prior to the commencement of the study. This study was approved by the Sandwell and West Birmingham Hospitals NHS Trust. Purposive sampling [22] was used in both the focus group and individual interviews and the researchers attempted to include participants with diverse demographic characteristics including age, sex, ethnicity and severity of incontinence.

The focus group was made up of eight individuals who were part of a local community continence user group. The group was used to discuss historical experiences of FI services and explore the views of people with incontinence on the design and implementation of the new ICP for FI (Additional file 1: Appendix 1- focus group topic guide). All participants gave informed written consent as per ethical approval. The focus group duration was 54 minutes.

Twenty patients entering into the new ICP were invited to take part in a qualitative study. Of these patients, five gave informed written consent to be interviewed for the purpose of the study. The participants interviewed had diverse characteristics, such as age, sex ethnicity and severity of FI. To explore patient experiences on this sensitive and potentially embarrassing topic we used narrative, face-to-face interviews [23] (Additional file 2: Appendix 2- interview topic guide). The interviews attempted to identify patient views on current FI service provision, the ease of accessibility to the services and their evaluation of the new ICP. Analysis was performed alongside data collection so that any emerging topics could be discussed in the subsequent interviews [24]. The five individual interviews had a mean duration of 37 minutes (range 24–51 minutes).

Both the interview and focus group data were recorded, transcribed and analysed by the same researcher (CR). The interview and focus group data was analysed using framework analysis [25, 26]. A separate independent analysis was performed for the interview data and focus group data, with the data subsequently being combined to form the results. Coding was performed in two cycles, firstly line-by-line initial coding with subsequent second cycle focused coding [27]. These codes related to the content of the transcripts alone. Consistency of coding and subsequent development of a thematic framework [26], was tested by two qualitative researchers (GD/CR) independently analysing the same transcripts. Discrepancies in coding were discussed and common themes were identified in both the patient interview and focus group data. The aim was to reach data saturation The numbers needed to do so can be variable [28], but following the focus group and individual interviews, no new themes were emerging and therefore the researchers concluded that the data saturation point had been reached.

## Results

Thirteen participants (8 focus group participants and 5 individual participant interviews) were interviewed between January and April 2013. All participants had FI (more than two incontinent episodes per month or inability to defer defecation for more than five minutes) with age, ethnicity and symptom severity varying considerably (Table 1).

**Table 1:** Participant characteristics

Characteristic	Value
Mean age, (range), years	63.5 (31–76)
Gender	
Female	11
Male	2
Ethnicity (n)	
Caucasian	9
Asian	3
Afro-Caribbean	1
Symptom severity- mean wexner score (range)	11.2 (6–20)
Patients who had sought help prior to interview/ focus group (not on ICP previously)	10
Patients who had received help prior to new ICP	8
Patients on ICP	5

Following the development of the thematic framework, four themes emerged with various sub themes being identified within these themes:

- Historical experience of access to continence services
- Historical evaluation of quality and provision of FI services
- Service users view on the redesign of the FI pathway
- Patient experience of the ICP pathway

These themes will be discussed below, with the use of sub themes helping to further focus the reporting of our results.

### Historical experience of access to continence services

#### Seeking help

Ten of the thirteen patients had received ‘traditional pathway’ treatment for their FI prior to the interview/focus group. All patients stated that they had eventually discussed their symptoms with their general practitioner (GP) or practice nurse, but did feel reluctant to do this openly due to embarrassment. Six patients noted that there had never been a direct enquiry from their GP regarding their FI symptoms.

*“Well erm yes, I had tried to get help but you know it's an embarrassing thing really, it’s not something that you want to discuss” Patient ID 4: Female, 65*

#### Referral

There was a strong sense that GPs were either unwilling to refer patients or that they were not aware of any services available. Five out of the thirteen patients were not referred to continence services at all, with eight patients experiencing a delay in being referred to the appropriate continence service, with three of them having to wait up to eighteen months. Four patients who were referred by their GPs mentioned that they were referred to various specialities within the hospital setting and not necessarily to the pelvic floor dysfunction service or the CCT.

*“I did go to see my GP, but I’m not sure he knew what to do with me really. It took a long time for me to be seen by someone who knew what they were going on about…. It probably took the best part of the year at least” Patient ID 2: Female, 52*

All participants felt that the continence services were almost “hidden” away. This was partly due to the fact that they presumed that the GPs did not know about the continence services and therefore could not refer. Lack of promotion or advertisement of services was postulated as a reason for this.

*“Yeah, I have been seen by a continence team and they certainly helped me but it took me a while to get there and to be honest I feel I could have been referred earlier but whether the GP didn't know about the service, I don't know” Patient ID 5: Female, 76*

### Historical evaluation of quality and provision of FI services

#### Experience of continence services

Eight participants had been assessed and managed by continence services in the past. Three participants had been managed by community continence services with the remaining five having been managed by various different continence services, ranging from community to hospital-based services. Common themes emerged in relation to all of the continence services. The participants felt that once they were in the service (see above) then their assessment was positive on the whole. Around half of the participants who had accessed services previously, mentioned that the healthcare professionals involved were comforting and responsive to their needs. However, some participants did mention that they had been referred to what felt like a “pad service”. Overall, the majority of participants who had received help prior to the introduction of the ICP felt that their previous assessments had been adequate whilst at the same time being appropriate in terms of sensitive enquiry into this embarrassing condition.

*“It was a few years ago now but I was referred to a continence service. But to be honest, all they did was give me pads, I got no other treatment. I just accepted it as I didn't know there was anything else available” Patient ID 9: Male, 75*

#### Management

Most participants were satisfied with how their symptoms were managed. However, the notion of satisfaction with the management they received was tempered by the delay in getting to that stage.

*“Yes, I think overall I was happy with my treatment but it didn't really last very long.” Patient ID 3: Female, 49*

*“Once I got to be seen by the nurse felt it was very good but there's no getting away from the fact that it took too long for me together and I suffered in that time” Patient ID 7: Female, 51*

Six of the participants felt that they were discharged from the continence services too early and decided against asking for another referral when their symptoms worsened, due to their initial difficulties in accessing the service and the belief that there may be nothing that could be done for them aside from pad provision.

### Service users view on redesign of the FI pathway

#### Service delivery

The focus group participants did not see any issues with the principle of being seen by a nurse initially as long as there is a “backup” readily available in the form of consultant review.

*“To be honest, and this is no disrespecting yourself, I'd prefer to see a nurse as generally they have a bit more time on their hands and are used to dealing with these things more often” Patient ID 2: Female, 52*

*“I have no issues seeing a nurse as long as if there are any problems I can get to see the doctor quickly” Patient ID 6: Female, 31*

#### Access to pathway

The focus group participants raised the potential issue of GPs being unaware of the new service and the potential need for promotion of the service throughout the local area to ensure that patients were referred appropriately and promptly to the service.

### Patient experience of the ICP

#### Location of care

The five participants who had experienced the new ICP pathway spoke positively about the location of care in the community setting.

*“Well I went to my GP and she just referred me straight in. She said she knew about this new service and that they’d see me at a local health centre. I didn’t have to wait long either which was a bonus, it was much quicker than when I was last referred” Patient ID 5: Female, 76*

All five participants had been triaged to the BFNS clinics but were at different stages within their assessment and management. One key element was the ease of access for patients at getting to appointments. This included the ease of parking, not having to pay for parking and being closer to home than the hospital trust. The participants mentioned that a potential issue could be for patients without their own modes of transport having to get to only two locations within a rather large geographical area. This was also a point raised by the focus group as a potential issue because patients with FI generally do not feel comfortable travelling long distances without easy access to a lavatory.

*“Yeah, yeah, it’s erm, it seems to be working well. This is my third visit and my incontinence is better. It’s good as well that it’s close to my house. Because I don’t have to traipse up to the hospital, it doesn’t cost me any money.” Patient ID 11: Male, 74*

*It’s fine for me because I live nearby. But if you live in a or b and you don’t have a car, it could take you a while to get here. I wouldn’t be so keen on that” Patient ID 10: Female, 73*

#### Healthcare professionals

The participants mentioned that all healthcare professionals they encountered within the service were knowledgeable and “put them at ease” by the fact that they seemed experienced when dealing with FI. All participants were happy to be seen by a nurse specialist rather than a consultant and did not see this as detrimental to their subsequent management. In fact, some participants actually preferred to see a nurse specialist as they were of the opinion that they could discuss more openly their problems with this individual.

#### Access to pathway

The five participants on the new ICP were referred via their GP promptly following the admission of their symptoms. They did not have to be reviewed by multiple services prior to being referred to the ICP for FI. All five participants had different GPs who were aware of the service. These participants did not wait longer than four weeks to be reviewed initially in the clinic.

#### Management and handheld document

All five participants at varying stages on the ICP were happy with the improvement in their symptoms so far. These participants had only received conservative management modalities but these alone were enough to improve their symptoms significantly. They found that the ICP handheld document was very useful for reminding them how to perform some of their conservative management exercises such as pelvic floor muscle exercises and correct defecatory dynamics. The handheld document was also very useful for them in that it allowed them to see how much their symptoms had improved and also to liaise with their GP or practice nurse with regards to what treatments they were currently undergoing.

*“So far my incontinence has got better. I can do things now that I couldn’t do before coming to see the nurses.” Patient ID 8: Female, 69*

*“The paperwork that I got sent was quite good actually, bit scary at first but the instructions on exercises turned out to be useful” Patient ID 4: Female, 65*

## Discussion

Historically, initial help seeking for patients with FI has been delayed along with subsequent referral. A new ICP was developed and the initial patient evaluation of it was positive. Service users made various suggestions how the FI pathway could have been improved. All participants involved in the study believed that the introduction of a new ICP for FI was a positive move. The issues that patients were most concerned about were access to continence services, GP awareness of continence services and prompt, effective management of their condition. These all appeared to be better in the new ICP. The participants who had been referred to the ICP reported having a very positive experience from referral to the commencement of management. This was mainly based around the qualities of the healthcare professionals involved and the availability of effective management techniques in a timely manner.

### Results in context

The NICE guidance and government documents identified issues with the delivery of continence services. The Good practice in continence services document [9] highlighted that there were a ‘number of problems across the country which affect access to and delivery of content and services’, of which they deemed identification; lack of involvement of users in service planning and delivery and geographical variations in numbers of staff, quality of service and waiting times to be the most troublesome. The Good practice in continence services document [9] was published in 2000, and further consolidated by guidance from the National Service Framework-for older people [10], but a recent broad scoping study of pelvic floor dysfunction (of which FI is a part) found current services to be characterised by fragmented approaches with asynchronous delivery, limited investment and poor inter-professional integration [11]. The authors argued that an improved service delivery model had the potential to improve outcomes through better inter-disciplinary collaboration and efficient use of resources.

The benefits of an integrated service for patients with FI are intended to include improved access to assessment, investigation and treatment with better and more acceptable treatment of symptoms (treatments themselves have not changed within this new ICP). A reduced number of hospital admission and re-admissions, with fewer outpatient appointments and direct access to appropriate secondary clinicians in secondary care would also benefit patients. Increased efficiency linked to appropriate therapeutic interventions, not just containment [8] would mean that symptom control is achieved quicker. There would be cost benefits to the wider economy with better healthcare utilisation, less job absenteeism and lower overall carer burden. The new ICP could give patients a quality of life that is at least equivalent to that offered by existing services for patients in terms of confidence, self-care and health maintenance.

This service was set up within the pelvic floor dysfunction unit with BFNS and an integrated community continence team. The authors are aware that this is not a standard service setup across the country and therefore this has to be taken into account. The majority of assessment and management could be provided by a GP and an adequately trained community continence nurse, with appropriate links and communication with a local/regional functional bowel Consultant for consideration of complex cases or neuromodulation and surgical procedures.

### Limitations of the study

FI is a potentially embarrassing and sensitive topic for patients. The fact that it may be uncomfortable for patients to talk about their condition may have led to potential bias when discussing their beliefs or experiences. Also, the fact that the main researcher was a clinician may also have caused patients to only discuss more positive aspects. It was made clear that CR did not have any vested interest in the service and was working to produce an honest evaluation of the service. As with most qualitative studies, our aim was to identify a range of experiences rather than define our participant sample as being representative. Our participant sample was diverse in the key characteristics but a longitudinal study may reveal further important aspects of an ICP for FI.

## Conclusion

The introduction of an ICP for FI appears beneficial to patients. However, this is an initial exploratory evaluation and therefore further work is needed before results are generalisable. A follow-up qualitative interview study will allow us to assess whether the current patient/participant concerns remain following the introduction of the ICP over a sustained period of time. In addition we will need to monitor the PROMS that are already in place within the ICP and compare them to the ‘traditional’ pathway outcomes. This will allow us to ensure that the improvement in patient symptoms, for patients referred to the ICP, is equal to or better than the ‘traditional’ pathway. The views of clinicians in primary and secondary care on the implementation of the ICP will also be investigated.

The introduction of an ICP for FI has the potential to solve some, if not all, of the issues related to the previously fragmented and disjointed continence services. However, important issues still remain regarding the effective promotion of the service to key stakeholders such as general practitioners, to ensure that appropriate patients are referred in a timely manner. Alongside this issue is the need for more community clinics in differing geographical areas to be available for patients to access. Patients identified shortcomings of the traditional pathway. It appears that service redesign has been possible and initial results suggest that patients could identify positive benefits from the new integrated care pathway.

## Additional files

Additional file 1: Appendix 1. Focus Group Protocol- protocol used for focus group discussion. (PDF 49 kb) Additional file 2: Appendix 2. Interview Topic Guide- topic guide used for individual interviews. (PDF 50 kb)

## Abbreviations

BFNS: Bowel function nurse specialist
CCT: Community Continence Team
FI: Faecal incontinence
GP: General practitioner
ICP: Integrated care pathway
NICE: National Institute for Clinical Health and Excellence
PROMS: Patient reported outcome measures
