# Supplementary material for: The design and initial patient evaluation of an integrated care pathway for faecal incontinence: a qualitative study
Source: BMC Health Serv Res. 2015 Oct 1;15:444. doi: 10.1186/s12913-015-1108-5 (PMC4589900; doi:10.1186/s12913-015-1108-5)
Supplement: Additional file 1: Appendix 1. — Focus Group Protocol- protocol used for focus group discussion. (PDF 49 kb) [file 12913_2015_1108_MOESM1_ESM.pdf]

Appendix 1

Focus Group Protocol

Introduction

Welcome. I want to thank you for coming today. My name is CR and I will be the facilitator for today's group discussion.

I have invited you to take part in this group discussion today because you are all members of the continence group and having met you I know that you are vocal and will give me your honest opinions.

What I learn from today's discussion will help me to improve faecal continence services in the region.

Ground rules

Before we begin, I would like to review a few ground rules for the discussion.

- I am going to ask you several questions; we do not have to go in any particular order but I do want everyone to take part in the discussion. I ask that only one person speak at a time.
- Feel free to treat this as a discussion and respond to what others are saying, whether you agree or disagree. I am interested in your opinions and whatever you have to say is fine with me. There are no right or wrong answers. I am just asking for your opinions based on your own personal experience. I am here to learn from you.
- Don't worry about having a different opinion than someone else. But please do respect each other's answers or opinions.
- If there is a particular question you don't want to answer, you don't have to.
- All answers will be treated as confidential. I shall not ask for anything that could identify you and we will only use first names during the discussion. I also ask that each of you respect the privacy of everyone in the room and not share or repeat what is said here in any way that could identify anyone in this room.
- I am tape recording the discussion today and also taking notes because I don't want to miss any of your comments. However, once I start the tape recorder I will not use anyone's full name and I ask that you do the same. Is everyone OK with this session being tape recorded?
- I will not include your names or any other information that could identify you in any reports we write. I will destroy the notes and audiotapes after I complete our study and publish the results.
- Finally, this discussion is going to take about 45-60mins

- **Does anyone have any questions before we start?**

Group Discussion

1. **What are your impressions of continence services in the region?**
   a. Community and secondary care
   b. Facilities
   c. Accessibility

2. **How would you improve the services?**
   a. What would be ideal?
   b. Change in location?
   c. Who would you prefer to see- consultant versus specialist nurses

3. **Given the change in faecal incontinence services proposed by Sandwell hospital and community services- what do you think will be the impact on patients accessing the service?**

Those are all of the questions that I wanted to ask.

Does anyone have any final thoughts that they haven't gotten to share yet?
