# Supplementary material for: The design and initial patient evaluation of an integrated care pathway for faecal incontinence: a qualitative study
Source: BMC Health Serv Res. 2015 Oct 1;15:444. doi: 10.1186/s12913-015-1108-5 (PMC4589900; doi:10.1186/s12913-015-1108-5)
Supplement: Additional file 2: Appendix 2. — Interview Topic Guide- topic guide used for individual interviews. (PDF 50 kb) [file 12913_2015_1108_MOESM2_ESM.pdf]

Appendix 2

Interview Topic Guide

Narrative interviews with opening question being question 1. Other questions can be used as guides for the rest of the conversation.

1. Tell me about your condition?
Onset, duration, effects on daily life

2. Can you talk me through the pathway of treatment for faecal incontinence?
How were you kept informed about your illness, treatment and logistics of treatment?

3. How many different health professionals have been involved in your care?
Number, roles, referred directly/separately?
Smooth pathway?
How long did you have to wait to see them?

4. How do you feel about the ease of access to the service?
Logistical/administrative ease

5. What is your impression of the service? How well is it working?
Has it helped? If so, how? If not, why not?

6. Are you aware of any changes planned in the near future for the faecal incontinence service?

7. In your opinion, how could the service be improved?
Appointments, logistics, diagnostics, treatments

8. What would count as success for you?
Clinical, lifestyle etc

Closure
Explain next steps in project/likely timescales for completion etc… Can you suggest anybody else that it might be helpful for us to interview? Thank you.
